# A Practical, Robust and Fast Method for Location Localization in Range-Based Systems

**DOI:** 10.3390/s17122869

**Published:** 2017-12-11

**Authors:** Shiping Huang, Zhifeng Wu, Anil Misra

**Affiliations:** 1 State Key Lab of Subtropical Building Science, South China University of Technology, Guangzhou 510640, China; ctasihuang@scut.edu.cn; 2 State Key Laboratory of Coal Resources and Safe Mining, China University of Mining and Technology, Xuzhou 221116, China; 3 School of Information, Guangdong Communication Polytechnic, Guangzhou 510650, China; wzf162200210@163.com; 4 Civil, Environmental and Architectural Engineering, The University of Kansas, Lawrence, KS 66045, USA

**Keywords:** location localization, indoor localization, positioning algorithm, Newton’s Method

## Abstract

Location localization technology is used in a number of industrial and civil applications. Real time location localization accuracy is highly dependent on the quality of the distance measurements and efficiency of solving the localization equations. In this paper, we provide a novel approach to solve the nonlinear localization equations efficiently and simultaneously eliminate the bad measurement data in range-based systems. A geometric intersection model was developed to narrow the target search area, where Newton’s Method and the Direct Search Method are used to search for the unknown position. Not only does the geometric intersection model offer a small bounded search domain for Newton’s Method and the Direct Search Method, but also it can self-correct bad measurement data. The Direct Search Method is useful for the coarse localization or small target search domain, while the Newton’s Method can be used for accurate localization. For accurate localization, by utilizing the proposed Modified Newton’s Method (MNM), challenges of avoiding the local extrema, singularities, and initial value choice are addressed. The applicability and robustness of the developed method has been demonstrated by experiments with an indoor system.

## 1. Introduction

The accurate localization of the objects is of importance in the information and artificial intelligence age [1,2]. Location localization technology is widely used in industrial and civil applications and has become a mainstay of geoscientific investigations [3,4,5,6,7,8]. These applications range from logistics to robot navigation, from security tracking to automotive safety, from medical services to house control and many other emerging wireless sensor network applications. Of these, the most popular daily example is Global Positioning System (GPS) [3,9]. The location accuracy of the GPS is typically in the range 3–10 m (median error about 2.9 m according to [10]) and it is sensitive to different areas such as open areas, half-open areas and urban canyons [10]. During the past decade or so, high demand has developed for more accurate localization in sparsely accessible environments. A number of technologies have been introduced for improving localization accuracy (up to centimeters) in the absence of the GPS, such as WLAN [11,12], Bluetooth [13,14], ZigBee [15,16], Ultrasound [17], GSM/CDMA [18,19], Acoustic Position System (APS) [20], and Ultra-Wide Band (UWB) technology [21,22,23,24]. The localization approaches are usually classified into three categories: (1) range-based [25], (2) angle-based [26] and (3) proximity-based [27]. Among these, range-based systems are considered to be more accurate [25].

A range-based system usually needs the installation of base stations (also known as anchors) at known locations. To determine the unknown position (tag) in a range-based system, we need to know the distances between the tag and the base stations. In particular, at least three independent distances are required to determine an unknown position in the planar system and four independent distances for an unknown position in the 3D system. To get the localization of the objects, it is necessary to solve a set of nonlinear equations. The solution of these nonlinear equations typically involves iteration to achieve convergence [28,29]. Consequently, choice of an improper algorithm can lead to undesirable computational cost, and more significantly, will lead to inaccurate positioning, especially in real time localization of moving objects, for which fast calculations are necessary.

Clearly, the accuracy of real time location localization is highly dependent on the efficiency of the solution algorithm for the nonlinear equations. Furthermore, the accuracy depends upon the quality of the distance measurements which can be affected by the clock drift [30], antenna delay [31,32], multipath [33,34], blockage [35] and interference [36]. A robust algorithm in this case should also be able to sift through bad or low-quality measurements. In this paper, we provide a novel efficient method for solving the nonlinear equations in range-based localization systems. The method is devised in a way that it can remove bad measurement data. In the following sections, we will describe the methodology, and then demonstrate through numerical examples the robustness and efficiency of the approach. The proposed location localization algorithm has been tested for an indoor localization system based upon UWB technology. The presented experiment demonstrates that the practical applicability of the algorithm, particularly for removing bad data for improving the accuracy.

## 2. Methodology

### 2.1. The Theory

To determine a location, a series of base stations are required. For example, the GPS system uses satellites as base stations. Each base station will emit messages to the unknown target tag. Based on the time of flight (TOF) between the station and the tag, the distance can be calculated by the propagation speed of the considered signal times the time of the flight. Therefore, we use a series of measured distance *r_mi_* to determine the position of the unknown tag. To this end, we are searching for the minimum of the following equation formed by considering the square of the difference of the measured and the actual distances between the tag and the base stations [37]:

(1) $${\sum f}_{i}=\sum {\left(\sqrt{{\left(x-x_{i}\right)}^{2}+{\left(y-y_{i}\right)}^{2}+{\left(z-z_{i}\right)}^{2}}-r_{mi}\right)}^{2}$$

In Equation (1), *i* is the number of the base station; *x*, *y*, *z* are the unknown position coordinates; and *x_i_*, *y_i_*, *z_i_* are the coordinates of the base stations. When *i* is larger than three for 2D localization (and four for 3D localization), the equation is over-determined. To obtain the extrema of Equation (1), it is to find the location where the derivatives are zero with respect to *x*, *y* and *z*, that is:

(2) $$F_{x}=\frac{\partial \sum f_{i}}{\partial x}=0;F_{y}=\frac{\partial \sum f_{i}}{\partial y}=0;F_{z}=\frac{\partial \sum f_{i}}{\partial z}=0$$

which represent a set of three nonlinear equations. Note that Equation (2) is a necessary but not a sufficient condition for finding an optimal solution strictly inside the considered domain. To solve Equation (2), we apply the iterative Newton’s Method [29,38,39]. These types of solution methods have been adapted in other localization schemes, such as those based upon the received signal strength (RSS) [29] or time difference of arrival (TDOA) [39]. It is also noteworthy that in many other localization problems both the tag-to-tag and tag-to-base station measurements are considered to minimize the position of the tags [40]. The presented approach, only considers tag-to-base station measurement and seeks to localize the location of tags individually. 

In the iterative Newton’s Method case, the solution at *j* + 1-th iteration can be updated using the following equation:

(3) [xj+1yj+1zj+1]=[xjyjzj]−[∂2∑fi∂x2∂2∑fi∂x∂y∂2∑fi∂x∂z∂2∑fi∂y∂x∂2∑fi∂y2∂2∑fi∂y∂z∂2∑fi∂z∂x∂2∑fi∂z∂y∂2∑fi∂z∂z]−1[∂∑fi∂x∂∑fi∂y∂∑fi∂z]

where *j* is the iteration number. To avoid the Gaussian elimination process [41,42], which can be time-consuming, it is efficient to utilize the explicit inverse form of Equation (3) given in the Appendix A. For demonstration of the presented approach, we will discuss the 2D localization in the following sections. Extension to the 3D localization is straightforward.

Although Newton’s Method is widely used due to its efficiency [7,43], there are three challenges when we use it to search for the minimum of a system: (1) choice of proper initial value for the first iteration can be obscure; (2) avoiding local extrema is usually difficult; and (3) singularity in Equation (3) will result the equations unsolvable. In order to solve the above problems and accelerate the iteration process, we first developed a geometric intersection model to narrow the search domain, which is the target search area. It is notable that each distance measurement *r_mi_* has some uncertain errors. We use Gaussian distribution [44] to describe the uncertainty as follows:

(4) $$f\left(r_{mi}\right)=\frac{1}{\sqrt{2\pi }\sigma_{i}}\exp\left(\frac{{\left(r_{mi}-r_{ai}\right)}^{2}}{2\sigma_{i}^{2}}\right)$$

where *f* is probability density function, *i* is the base station number, *r_m_* is the measurement value, *r_a_* is the actual distance between the station and the unknown tag, and *σ_i_* is the standard deviation which depends upon the sensor technology and the environment. The uncertainty or random variations arise from a variety of sources, such as antenna error, system drift, multi-hops and so on. For practical computations, measurement errors are considered in a finite range, which covers a large but less than 100% probability. The assumption is that the measured distance lies in the following range:

(5) $$r_{a}{}_{i}-k\sigma_{i}<r_{mi}<r_{a}{}_{i}+k\sigma_{i}$$

where *k* is a positive real number. Thus, the errors present in the distance measurement, whether they arise from multi-hops or other factors, are incorporated in the proposed localization algorithm as the probability measure *k*. The probability can be obtained by the integration the probability density function as follows:

(6) $$P\left(r_{ai}-k\sigma_{i}<r_{mi}<r_{ai}+k\sigma_{i}\right)=\int_{r_{ai}-k\sigma_{i}}^{r_{ai}+k\sigma_{i}}\frac{1}{\sqrt{2\pi }\sigma_{i}}\exp\left(\frac{{\left(r_{mi}-r_{ai}\right)}^{2}}{2\sigma_{i}^{2}}\right)dr_{mi}$$

**Figure 1:**
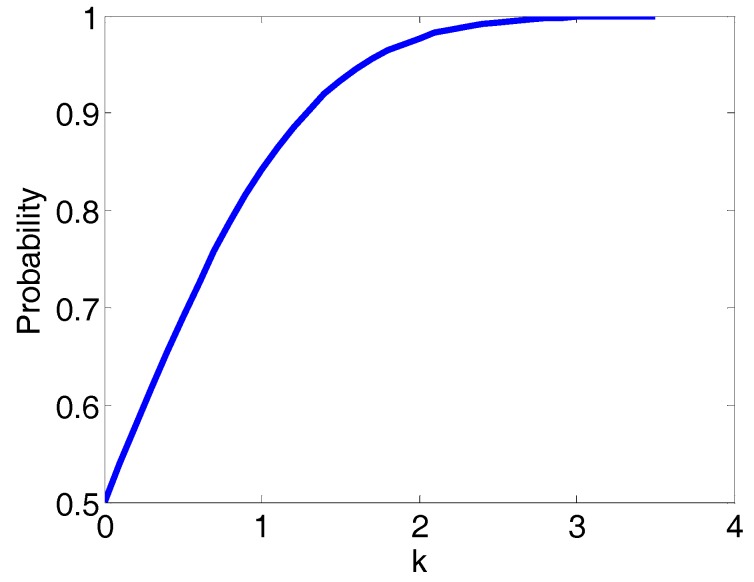
The probability of *P*(*r_ai_* − *kσ_i_* < *r_mi_* < *r_ai_* + *kσ_i_*) with different *k*.

We know when the parameter *k* = 1, 2, 3, the probability of the measured distance occurring in the corresponding ranges will be 68%, 95% and 99.7%, as seen in Figure 1.

Now, we consider the probability for the actual distance located in a specific circular area. The area is formed with the center of station *i* and radius of *r_i_*, where *r_i_* is the upper bound distance defined as $r_{i}=r_{mi}+k\sigma_{i}$. The probability that the unknown tag lies in the circular area (with the center of station *i* and radius of *r_i_*) is *P*(*r_mi_* < *r_ai_* + *kσ_i_*). The unknown tag should be located inside all similar circular areas centered at the various stations. Clearly, therefore, the unknown tag should reside inside the intersection of these circle areas. The equation for the circle’s intersection can be described as:

(7) $$\left[{\left(x-x_{1}\right)}^{2}+{\left(y-y_{1}\right)}^{2}<{\left(r_{1}\right)}^{2}\right]\cap \left[{\left(x-x_{2}\right)}^{2}+{\left(y-y_{2}\right)}^{2}<{\left(r_{2}\right)}^{2}\right]\ldots \cap \left[{\left(x-x_{n}\right)}^{2}+{\left(y-y_{n}\right)}^{2}<{\left(r_{n}\right)}^{2}\right]$$

**Figure 2:**
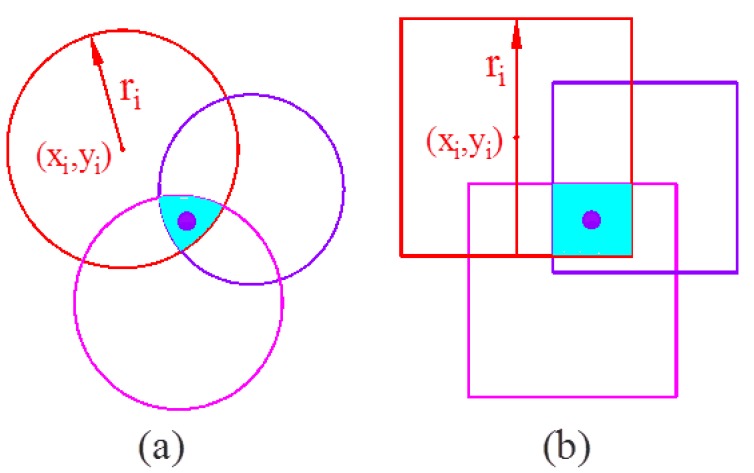
The intersection process: (**a**) circle case, (**b**) square case.

Figure 2a represents the intersection of three circle with radii *r_i_* and the blue area is the overlap area, where the unknown position is located. Geometric constraints imposed by circle-circle intersection has been used widely, see for example [45,46,47]. In the proposed algorithm, for computational simplicity and efficiency, we utilize a square centered at a station for which the equation for intersection is given as:

(8) $$\left[x<\left|x_{1}-r_{1}\right|\cap y<\left|y_{1}-r_{1}\right|\right]\cap \left[x<\left|x_{2}-r_{2}\right|\cap y<\left|y_{2}-r_{2}\right|\right]\ldots \cap \left[x<\left|x_{n}-r_{n}\right|\cap y<\left|y_{n}-r_{n}\right|\right]$$

It is noted that the blue overlap area of the square case (Figure 2b) is larger than that of the circle case. However, in the square case, we can efficiently perform the intersection calculation mathematically. A key advantage for intersection using squares is that multiple intersections can be processed by applying the procedure in Equations (8) through (12) in a repetitive manner. In contrast, for circular geometry only two circle intersection can be considered at a time and determination of overlap of multiple circles becomes cumbersome.

**Figure 3:**
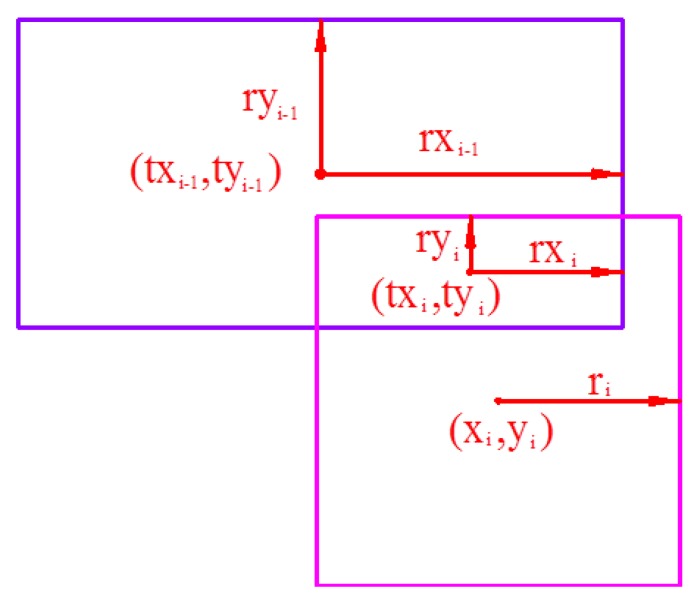
Iteration process of the square intersection.

The computational algorithm begins by considering the intersection of square areas of any two arbitrarily chosen stations. The overlap area generated by these will subsequently interact with the square area of the third station and so on. At the end of each intersection computation, a new overlap rectangular area will be generated with the centroid (*tx_i_*, *ty_i_*). The new rectangle generated by the intersection of the rectangle (*tx_i_*_−1_, *ty_i_*_−1_) and square (*x_i_*, *y_i_*), is observed in Figure 3, where the coordinates of the rectangular centroid *tx_i_* and *ty_i_* can be obtained by:

(9) $$tx_{i}=\frac{1}{2}\left(\min(tx_{i-1}-rx_{i-1},x_{i}-r_{i})+\min(tx_{i-1}+rx_{i-1},x_{i}+r_{i})\right)$$

(10) $$ty_{i}=\frac{1}{2}\left(\min(ty_{i-1}-ry_{i-1},y_{i}-r_{i})+\min(tx_{i-1}+rx_{i-1},y_{i}+r_{i})\right)$$

and the rectangle’s half length (*rx_i_*) and half width (*ry_i_*) and can be described as:

(11) $$rx_{i}=\frac{1}{2}\left(\min(tx_{i-1}+rx_{i-1},x_{i}+r_{i})-\min(tx_{i-1}-rx_{i-1},x_{i}-r_{i})\right)$$

(12) $$ry_{i}=\frac{1}{2}\left(\min(ty_{i-1}+ry_{i-1},y_{i}+r_{i})-\min(ty_{i-1}-ry_{i-1},y_{i}-r_{i})\right)$$

Once the target search area is obtained, we can use two methods to search the minima inside the area. The first method is the Direct Search Method (DSM). We mesh the target area into a series of discrete points and calculate each point’s value using Equation (1). Comparing these values, we can find the minimum, which is then taken as the solution. The DSM is useful when the target domain is small or for the coarse localization. This method is very simple to implement, however can lead to inefficiencies and loss of accuracy when the target domain is large. 

The second method is the Modified Newton’s Method (MNM). In this method, we mesh the target search area into predetermined discrete points, usually 2*n* discrete points. We then use each point as our initial value for the Newton’s iteration given in Equation (3). It is notable that the solution of Equation (3) can be found in closed form as given in Appendix A, since the problem we are solving involves a matrix of rank 3 (2 in 2D). The approach is completely different from those discussed in other localization schemes that use the modified Newton’s method, where the difficulty arises from non-positive definiteness of the Hessian matrix and involves either the simultaneous consideration of several sensors and targets [29] or TDOA [39]. In our case, instead of determination of positive definiteness of the Hessian matrix, we only need to guarantee *cc* (the closed form is given in the Appendix A) is a non-zero number. If *cc* is equal to zero, we will give up the Newton’s iteration based on the corresponding guess. From these Newton method’s solutions, we numerically determine the ‘global minimum’. It is noted that the numerically determined ‘global minimum’ may not strictly coincide with the absolute or the smallest overall value of the function but is closest to it. 

Furthermore, the reliability of the unknown tag located in the target area depends on the variance of the measurement *r_mi_*. Each distance reported by the station is independent, thus the probability of the unknown tag located in the final target area will be:

(13) $$\begin{array}{l} P(r_{m1}<r_{a1}+k\sigma_{1},r_{m2}<r_{a2}+k\sigma_{2}\ldots r_{mn}<r_{an}+k\sigma_{n})\\ =P(r_{m1}<r_{a1}+k\sigma_{1})\;p(r_{m2}<r_{a2}+k\sigma_{2})\ldots p(r_{mn}<r_{an}+k\sigma_{n}) \end{array}$$

**Figure 4:**
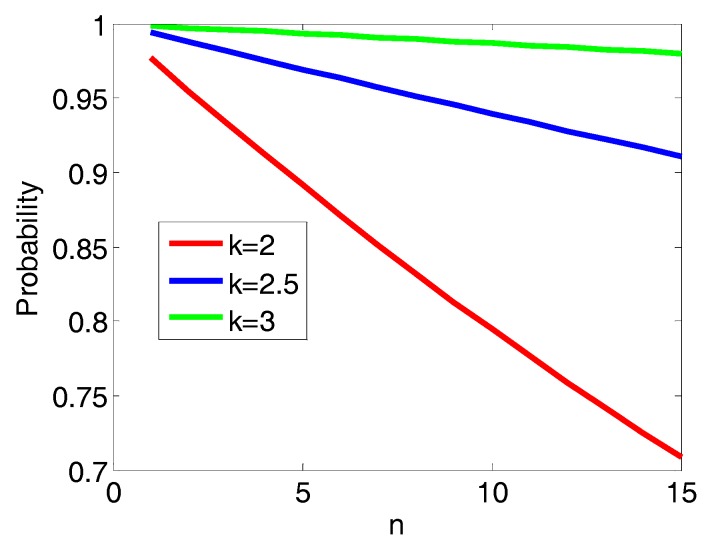
The probability of *P*(*r_mi_* < *r_ai_* − *kσ_i_*) with different *k* and *n*.

It is noted that the probability above is for the circle’s intersection. Since we enlarge the circle to square so the probability for the square’s intersection is larger than that of the circle’s intersection. Since the upper bound distance is directly related to the parameter *k*, a large value of *k* may result in a larger search domain and will lead to a higher probability of the unknown tag located in the search domain. Figure 4 demonstrates that the probability *P* increases as *k* increases, while *P* decreases as *n* increases. On the other hand, smaller *k* indicates more stringent screening criteria with the measurements data, which is discussed in the self-correcting process. The optimal solution is expected to be contained within the intersections of all the areas formed by the upper bound distances. For example, if a particular measurement does not have an intersection while other stations have intersections, the measurement is deemed as a bad point and discarded. 

### 2.2. Self-Correcting Process

Low-quality measurements will definitely lead to inaccurate results. In this section, we will demonstrate how to remove bad measurements. As observed in Figure 4, as *n* increases, for a given *k*, fewer measurement meet the high-quality criterion. Furthermore, as parameter *k* decreases for given *n*, also fewer measurements meet the high-quality criterion. These low-quality data should be removed. As discussed above, the error bounds are set to be within the range (*r_ai_ − kσ_i_* < *r_mi_* < *r_ai_* + *kσ_i_*). When *r_mi_* < *r_ai_* − *kσ_i_*, the unknown point may be outside the intersection region. In this case, the intersection is an empty set and we have to discard the bad points. In the algorithm, if a square area associated with a station has no intersection with the overlap area (as given in Figure 3), the measurement associated with this station is deemed as a bad point and discarded. However, when *r_mi_* > *r_ai_ + kσ_i_*, the geometric intersection works. Therefore, to make sure that the intersection domain is within the range *r_ai_ − kσ_i_* < *r_mi_* < *r_ai_* + *kσ_i_*, for each intersection domain generated by Equations (8)–(12), the distance between the station and the intersection domain should be within the error bounds. Otherwise the measurement should be removed. The upper bound calculated distance *cru_i_* between the station(*x_i_*,*y_i_*) and the intersection domain with the centroid (*tx_i_*, *ty_i_*) can be given by:

(14) $$cru_{i}=\sqrt{{\left(\left|x_{i}-tx_{i}\right|+rx_{i}\right)}^{2}+{\left(\left|y_{i}-ty_{i}\right|+ry_{i}\right)}^{2}}\geq r_{mi}-k\sigma_{i}$$

The lower bound calculated distance *crl_i_* between the station and the intersection domain can be given by:

(15) $$crl_{i}=\sqrt{\min{\left(x_{i}-x(x\subset [tx_{i}-rx_{i},tx_{i}+rx_{i}])\right)}^{2}+\min{\left(y_{i}-y(y\subset [ty_{i}-ry_{i},ty_{i}+ry_{i}])\right)}^{2}}\leq r_{mi}+k\sigma_{i}$$

### 2.3. The Complete Algorithm

**Figure 5:**
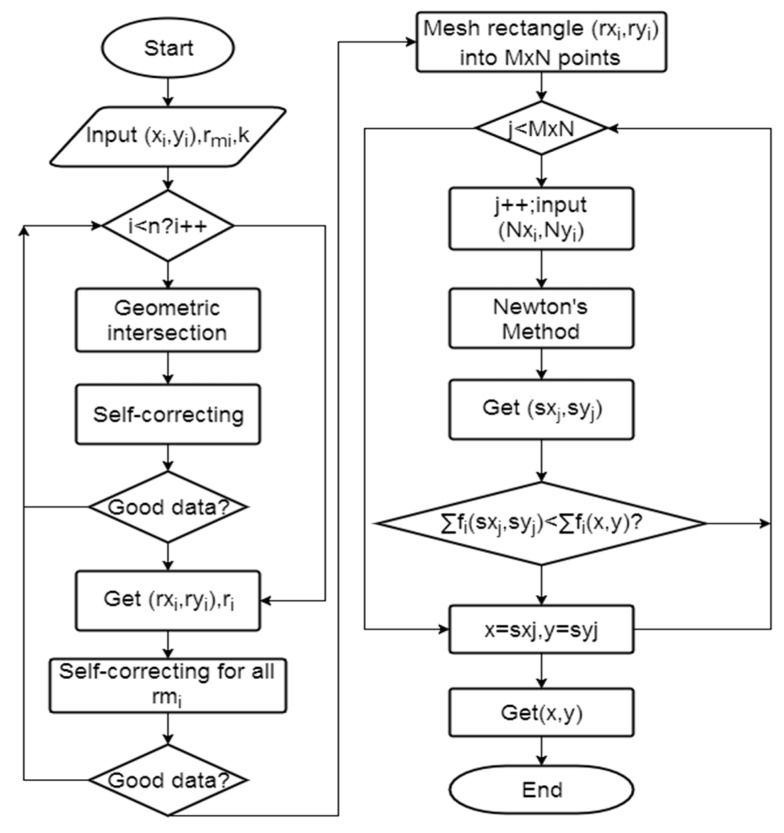
Flowchart of the computational algorithm.

The computational algorithm based upon MNM can be summarized as follows. A flow chart of the algorithm can be seen in Figure 5.

Step 1: Input the station coordinates (*x*_i_, *y*_i_) and distance *r_mi_*, *k*.
Step 2: Perform the geometric intersection algorithm based on Equations (8)–(12). Each iteration of algorithm will generate a new intersection region. The new region will intersect with the measurement square until all the stations are exhausted.
Step 3: Using the self-correcting process to determine whether the current measurement is a low-quality measurement. If it is a low-quality measurement, then remove this data and go back to step 1.
Step 4: When the target search domain obtained, do the self-correcting process again to check whether any low-quality measurement still exists. Those low-quality measurements should be removed and the geometric intersection restarted until no measurement is considered as low-quality measurement.
Step 5: Mesh the intersection region into *M* × *N* points (usually *M* × *N* = 2*n*). Use the mesh grid coordinates as the initial guess of Newton’s Method.
Step 6: Use Newton’s iterations to refine the potential unknown point (*sx_i_*, *sy_i_*), which could be one of the local minimums.
Step 7: Find the ‘global minimum’ by comparing Ʃ*f_j_*(*sx_j_*, *sy_j_*) and Ʃ*f_j_*(*x*, *y*). If Ʃ*f_j_*(*sx_j_*, *sy_j_*) < Ʃ*f_j_*(*x*, *y*), substitute *x* = *sx_i_*, *y* = *sy_i_*.
Step 8: Repeat step 7 for all the mesh grid points.
Step 9: Get the minimum Ʃ*f*(*x*, *y*), and (*x*, *y*) is the solution.

Since the minimization is unconstrained, it is possible that the computed solution ends outside of the search domain. In the present algorithm, if the solution is outside the search domain, we will update the starting points and perform the calculation again as detailed in the flowchart, till we get the desired result. 

## 3. Results and Discussion

### 3.1. A Numerical Example

A numerical example has been designed to demonstrate the applicability of the proposed approach. Here, MATLAB is used to conduct numerical analysis in Windows 7. The computer configuration is as follows: the processor is an Intel i7 7400, memory 8 GB, disk 500 GB. At first seven base stations are generated randomly in the 100 m × 100 m square area. The coordinates of the stations are listed in Table 1. Then an unknown tag position is generated randomly inside the 100 m × 100 m square area. The measurement is assigned as the real distance between station and the tag plus a random variance of the Gaussian distribution, as shown in Table 1.

**Figure 6:**
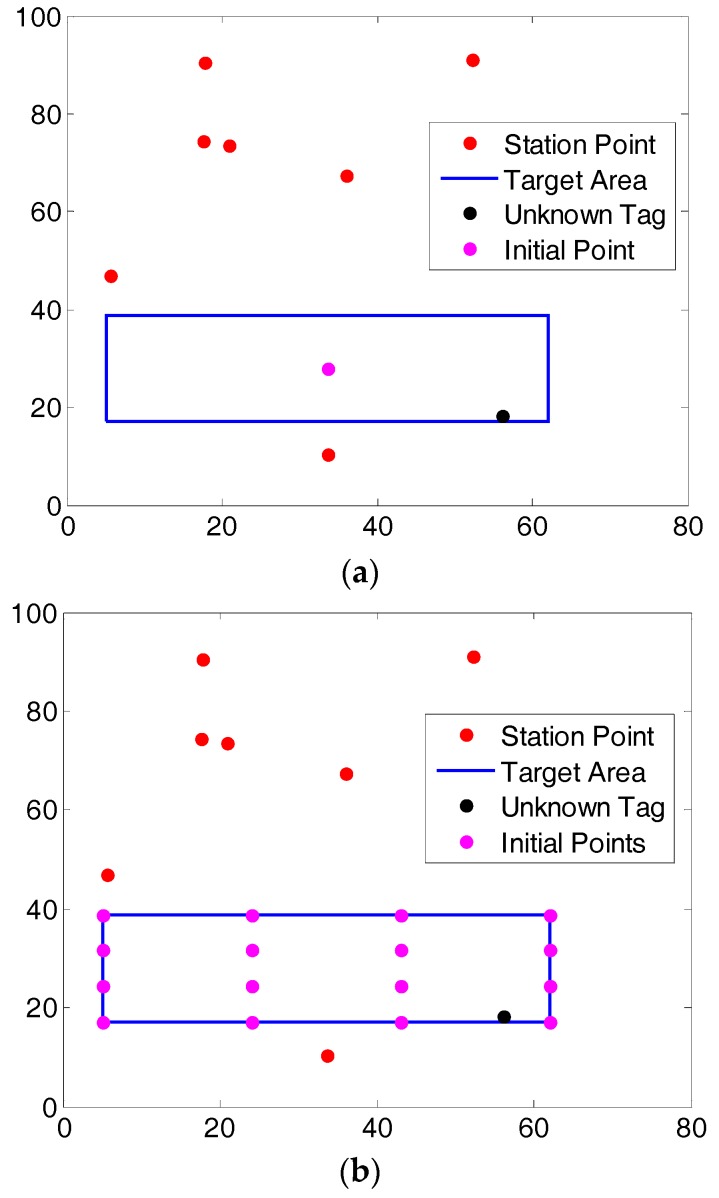
Layout of the station and initial search points (in m): (**a**) single initial guess, (**b**) 16 initial guesses.

In Figure 6, the red points represent the positions of the 7 base stations; the black point is the unknown tag; the blue rectangle is the target area and purple points are the locations of initial guess. In Figure 6a we have only 1 initial guess; while in Figure 6b we have 16 initial guesses. 

The choice of a proper number of the initial guess is very important. Usually inside the target area there may be several local extrema. To obtain the correct location, we have to make sure that we have searched all of these local extrema. However, choosing a larger number of initial points will result in increased computation time. Up to *2n* number of initial guess may be selected in order to converge to the correct location in the shortest possible time. Mathematically, for *n* station system, there are at most 2*n* extrema (*n* minimums and *n* maximums). However, this cannot guarantee that with 2*n* initial guesses we can search all the extrema since different initial guesses can converge to the same extrema. In practice, usually 2*n* initial guesses in the target search area are enough to get all the extrema.

**Figure 7:**
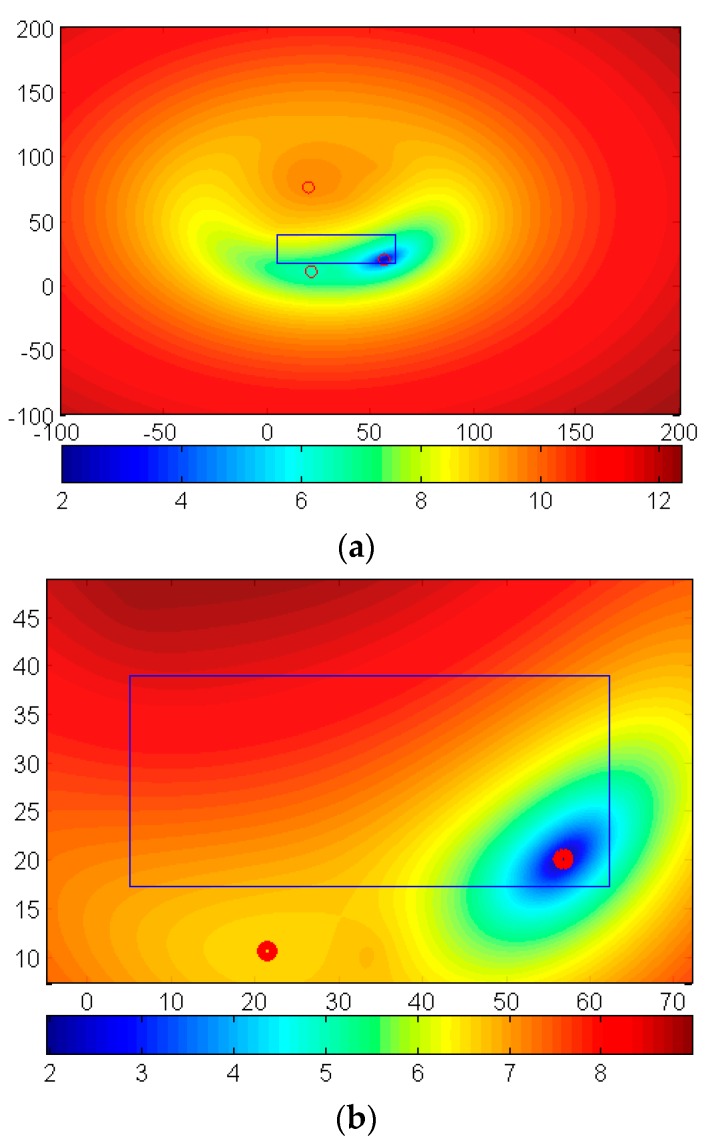
The contour of the logarithm of Equation (1) and the target area (in m): (**a**) contour of 200 m × 200 m square domain, (**b**) contour of 70 m × 45 m square domain.

In Figure 7, the contour of the logarithm of Equation (1) is plotted based on the data in Table 1. It can be seen from Figure 7a, that the ‘global minimum’ is difficult to detect due to the presence of many closely spaced local minima that are within the calculation error. The regular algorithm, in this case, is unable to distinguish amongst these minima and converges arbitrarily to one of the many minima. Clearly, in this case, the traditional Newton’s Method can be risky, since there is no way to know the ‘global minimum.’ On the other hand, in the proposed method, whose results are shown in Figure 7a, we can see that we can localize the minimum inside the target area. Remark that the contours in Figure 7b are over a subarea of Figure 7a and the ‘global minimum’ is clearly detectable. However, if the initial values are not chosen properly, we will not get the minimum inside the target area. It is observed in Figure 7b that one initial guess will be very risky because they may converge to the location outside the target region, which is the reason why we need multiple initial guesses in the MNM model.

### 3.2. Comparison of Calculation Time between Direct Search Method and Modified Newton’s Method

**Figure 8:**
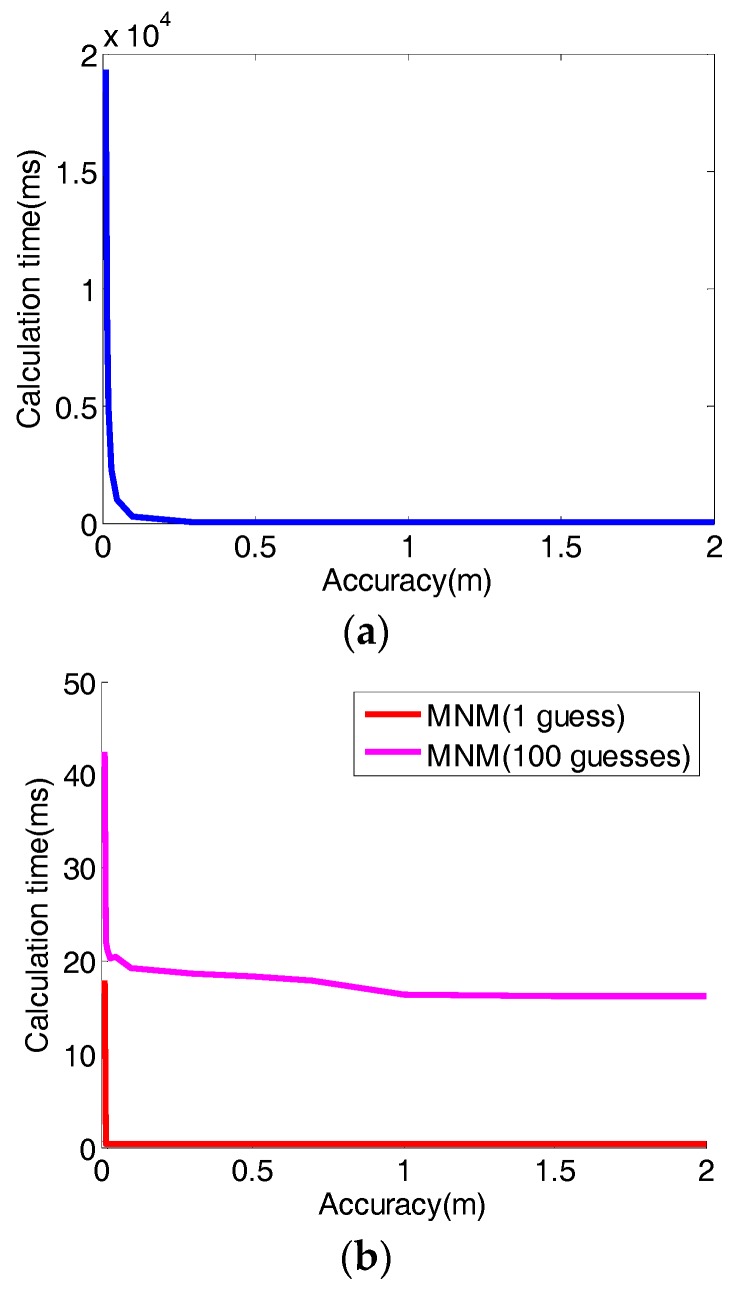
Computation time using different models: (**a**) Direct Search Model (DSM), (**b**) Modified Newton’s Method (MNM).

In addition to the difficulty associated with the detection of ‘global minimum,’ the calculation time of the Direction Search Method and the Modified Newton’s Method can be vastly different. When the accuracy is high, the direct search method’s calculation can be orders of magnitude more expensive than that of the Modified Newton’s Method, as shown in Table 2 and Figure 8. Furthermore, the comparison of 1 initial point and 100 initial points also can be observed in Table 2 and in Figure 8b. It is remarkable that for the case of accuracy = 0.01 m, when we use 100 initial guess points for the MNM, which is far more initial points than we really need, the computational time is only 1/454 of the time using Direct search method. It is noted that here the accuracy means the accuracy of the approximation of the function to be optimized.

### 3.3. Effect of Low-Quality Data

**Figure 9:**
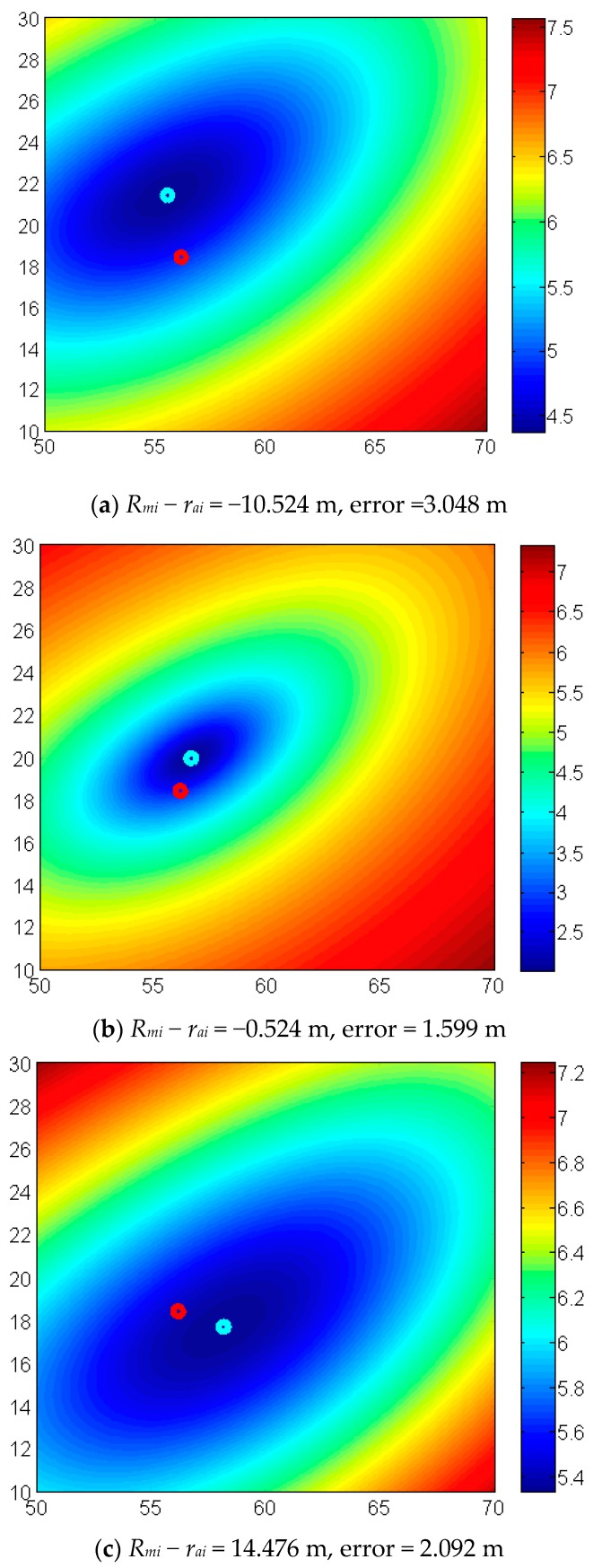
The contour of the logarithm of Equation (1) with different fluctuation of measurements (form 55 m to 80 m) in station #7. The error in (**a**–**c**) refers to location error.

As we discussed above, the low-quality measurement can lead to inaccurate results. To demonstrate the effect of low quality data, we modified the measurement reported by station 7 in Table 1 to a series of different values ranging from 55 m to 80 m. Note that the actual distance from station 7 to the tag is 65.524 m. As shown in Figure 9, the red point is the actual location while the green point is the solution obtained by the proposed MNM. It is observed that the low quality data affects the localization in a manner depending upon the location and the error of all the measuring distances. In general, larger measurement error will lead to more inaccurate results. 

### 3.4. Comparison of Modified Newton’s Method and traditional Newton’s Method

**Figure 10:**
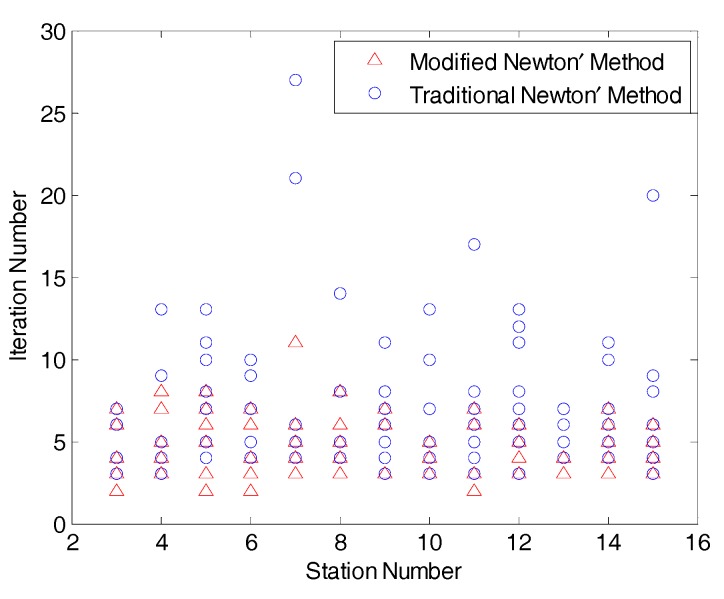
Comparison of maximum iteration between Modified Newton’s Method and traditional Newton’s Method for different stations.

Even for the case that there is an easily identifiable ‘global minimum’ (that is, the solutions do not have closely spaced local minima), the MNM method, with a defined target area, can be vastly efficient. The target area allows us to select a better initial guess and thus the convergence process of the Modified Newton’s Method is much faster than traditional Newton’s method (where the initial value is randomly generated), as in [28]. To illustrate the efficiency, we generated large experimental data, where the number of the station ranged from 3 to 15. For each case of same number of stations, ten different layouts were randomly generated, to obtain altogether 130 configurations. The localization convergence (for accuracy to 0.001 m) results are shown in Figure 10. We can observe that the Modified Newton’s Method can be considerably faster than the traditional Newton’s method. The total iteration number for MNM are found to be at most only 70% of the traditional Newton’s Method.

### 3.5. An Indoor Localization Experiment

**Figure 11:**
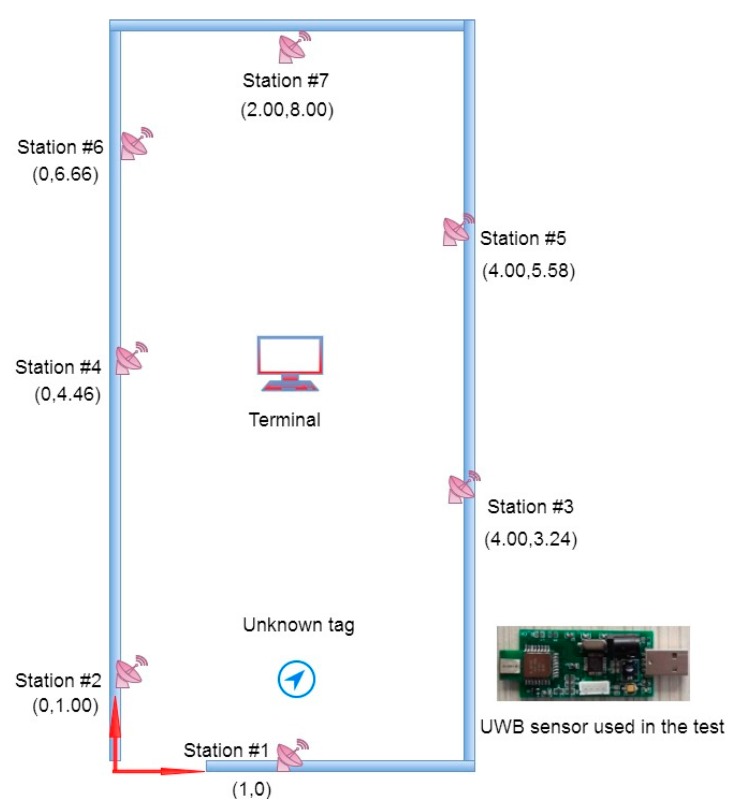
The experimental design.

Recently, UWB technology has attracted lots of attention for indoor localization. We here use our localization tag and anchors based on an UWB transceiver. The experiment is carried in our lab, where the size is 8 m × 4 m. We installed seven anchors (stations) to determine the location (unknown tag), as shown in Figure 11. The measurement accuracy for the UWB has been reported to be 0.30 m [4]. In the measurements observed in our system, the variance of the measured distance was found to range from 0.04 m to 0.20 m based upon 500 repeats. In the algorithm, we take *σ_i_* to be 0.3 m, as shown in Table 3. 

The data used as input for the algorithm is listed in Table 3 In the self-correcting process, the measurement at station 6 was removed. The solution (2.11 m, 0.68 m) has 0.33 m error compared to the independently measured location (2.00 m, 1.00 m). Without the self-correcting process, the solution (2.21 m, 0.55 m) has an error of 0.49 m. This demonstrates the improved accuracy due to the presented algorithm.

## 4. Conclusions

In this paper, we provide a novel approach to efficiently solve the minimization of nonlinear localization equations. The approach can simultaneously eliminate the bad measurement data in range-based systems. The approach can be extended to constrained minimization problem with a predefined search domain. In this approach, a geometric intersection model was developed to narrow the target search area, where Newton’s Method and the Direct Search Method are used to search for the unknown position. Not only does the geometric intersection model offer a small bounded search domain for Newton’s Method and the Direct Search Method, but also it can self-correct the bad measurement data, which is particularly useful when dealing with large overdetermined systems. The Direct Search Method is useful for the coarse localization or small target search domain, while the Newton’s Method can be used for accurate localization. For accurate localization, by utilizing the proposed Modified Newton’s Method (MNM), challenges of avoiding the local extrema, singularities, and initial value choice are addressed. Through example calculations, the proposed model is shown to be robust, accurate and efficient. The applicability and robustness of the developed method has been demonstrated by experiments with an indoor UWB system. The proposed method can be used for accurate indoor and outdoor localization.

## Figures and Tables

**Table 1:** The base station coordinate and distance measurement to the tag (*k* = 2.5, *P* = 96%, *σ_i_* = 1 m).

Station, *n*	1	2	3	4	5	6	7
Coordinate *x_i_* (m)	17.812	35.963	5.670	52.189	33.585	17.567	20.895
Coordinate *y_i_* (m)	90.515	67.539	46.847	91.213	10.401	74.555	73.627
measurement *r_mi_* (m)	82.159	52.119	56.136	71.467	25.956	65.994	64.236
measurement errors (m)	0.486	1.000	1.829	1.437	1.975	2.143	1.288

**Table 2:** The calculation time (ms) using different methods (*k* = 2.5, *P* = 96%, *σ_i_* = 1 m).

Accuracy (m)	0.01	0.015	0.02	0.03	0.05	0.1	0.3	0.5	0.7	1	1.5	2
DSM time	19309	8570	4954	2274	975	282	26.04	8.96	4.20	2.05	0.93	0.56
MNM time (initial guess = 1)	17.96	0.44	0.42	0.41	0.40	0.40	0.39	0.37	0.36	0.39	0.36	0.32
MNM (initial guesses = 100)	42.47	22.02	21.14	20.32	20.47	19.26	18.61	25.33	17.88	16.32	16.29	16.26

**Table 3:** The experimental data for the indoor experiment (*k* = 2.5, *P* = 96%, *σ_i_* = 0.3 m).

Station, *n*	1	2	3	4	5	6	7
Coordinate *x_i_* (m)	2.00	0.00	4.00	0.00	4.00	0.00	2.00
Coordinate *y_i_* (m)	0.00	1.00	3.24	4.46	5.58	6.66	8.00
Measurement *r_mi_* (m)	1.22	2.12	3.25	4.36	5.39	7.01	7.62
Actual distance (m)	1.00	2.00	3.01	4.00	5.00	5.98	6.99

## Appendix A

The explicit form of Newton’s Method for Equation (3) in a 3D case is as given below:

(A1) [xj+1yj+1zj+1]=[jxyjzj]−[∂2∑fi∂x2∂2∑fi∂x∂y∂2∑fi∂x∂z∂2∑fi∂y∂x∂2∑fi∂y2∂2∑fi∂y∂z∂2∑fi∂z∂x∂2∑fi∂z∂y∂2∑fi∂z∂z]−1[∂∑fi∂x∂∑fi∂y∂∑fi∂z]=[jxyjzj]−[MxxMxyMxzMyyMyzMzz]−1[MxMyMz]=[jxyjzj]−[K11K12K13K22K23K33][MxMyMz]

where:

$$S_{i}=\sqrt{{\left(x-x_{i}\right)}^{2}+{\left(y-y_{i}\right)}^{2}+{\left(z-z_{i}\right)}^{2}}$$

$$M_{x}=\sum_{i=1}^{n}\frac{2(S_{i}-r_{m}{}_{i})(x-x_{i})}{S_{i}}$$

$$M_{y}=\sum_{i=1}^{n}\frac{2(S_{i}-r_{m}{}_{i})(y-y_{i})}{S_{i}}$$

$$M_{z}=\sum_{i=1}^{n}\frac{2(S_{i}-r_{m}{}_{i})(z-z_{i})}{S_{i}}$$

$$M_{xx}=\sum_{i=1}^{n}\frac{2}{S_{i}{}^{3}}(-{\left(y-y_{i}\right)}^{2}-{\left(z-z_{i}\right)}^{2}-S_{i}(2xx_{i}+2yy_{i}+2zz_{i}-x_{i}{}^{2}-y_{i}{}^{2}-z_{i}{}^{2}-x^{2}-y^{2}-z^{2}))$$

$$M_{yy}=\sum_{i=1}^{n}\frac{2}{S_{i}{}^{3}}(-{\left(x-x_{i}\right)}^{2}-{\left(z-z_{i}\right)}^{2}-S_{i}(2xx_{i}+2yy_{i}+2zz_{i}-x_{i}{}^{2}-y_{i}{}^{2}-z_{i}{}^{2}-x^{2}-y^{2}-z^{2}))$$

$$M_{\mathrm{zz}}=\sum_{i=1}^{n}\frac{2}{S_{i}{}^{3}}(-{\left(x-x_{i}\right)}^{2}-{\left(y-y_{i}\right)}^{2}-S_{i}(2xx_{i}+2yy_{i}+2zz_{i}-x_{i}{}^{2}-y_{i}{}^{2}-z_{i}{}^{2}-x^{2}-y^{2}-z^{2}))$$

$$M_{xy}=\sum_{i=1}^{n}\frac{2(x-x_{i})(y-y_{i})r_{m}{}_{i}}{S_{i}{}^{3}}$$

$$M_{xz}=\sum_{i=1}^{n}\frac{2(x-x_{i})(z-z_{i})r_{m}{}_{i}}{S_{i}{}^{3}}$$

$$M_{yz}=\sum_{i=1}^{n}\frac{2(y-y_{i})(z-z_{i})r_{m}{}_{i}}{S_{i}{}^{3}}$$

and:

$$K_{ij}=M_{ij}{}^{-1}=\frac{1}{cc}\left[\begin{array}{ccc} M_{yy}M_{zz}-M_{yz}{}^{2} & M_{xy}M_{zz}-M_{xy}M_{yz} & M_{xy}M_{zz}-M_{xy}M_{yz}\\ & M_{zz}M_{xx}-M_{xz}{}^{2} & M_{xx}M_{yz}-M_{xy}M_{xz}\\ & & M_{yy}M_{xx}-M_{xy}{}^{2} \end{array}\right]$$

$$cc=2M_{xz}M_{xy}M_{yz}-M_{xz}{}^{2}M_{yy}+M_{xy}{}^{2}M_{zz}-M_{yz}{}^{2}M_{xx}+M_{xx}M_{yy}M_{zz}$$

Similarly, the explicit form of Newton’s Method for Equation (3) in 2D case is as below:

(A2) [xj+1yj+1]=[xjyj]−[∂2∑fi∂x2∂2∑fi∂x∂y∂2∑fi∂y∂x∂2∑fi∂y2]−1[∂∑fi∂x∂∑fi∂y]=[xjyj]−[MxxMxyMyy]−1[MxMy]=[xjyj]−[K11K12K22][MxMy]

where:

$$S_{i}=\sqrt{{\left(x-x_{i}\right)}^{2}+{\left(y-y_{i}\right)}^{2}}$$

$$M_{x}=\sum_{i=1}^{n}\frac{2(S_{i}-r_{m}{}_{i})(x-x_{i})}{S_{i}}$$

$$M_{y}=\sum_{i=1}^{n}\frac{2(S_{i}-r_{m}{}_{i})(y-y_{i})}{S_{i}}$$

$$M_{xx}=\sum_{i=1}^{n}\frac{2}{S_{i}{}^{3}}(-{\left(y-y_{i}\right)}^{2}-S_{i}(2xx_{i}+2yy_{i}-x_{i}{}^{2}-y_{i}{}^{2}-x^{2}-y^{2}))$$

$$M_{yy}=\sum_{i=1}^{n}\frac{2}{S_{i}{}^{3}}(-{\left(x-x_{i}\right)}^{2}-S_{i}(2xx_{i}+2yy_{i}-x_{i}{}^{2}-y_{i}{}^{2}-x^{2}-y^{2}))$$

$$M_{xy}=\sum_{i=1}^{n}\frac{2(x-x_{i})(y-y_{i})r_{m}{}_{i}}{S_{i}{}^{3}}$$

$$K_{ij}=M_{ij}{}^{-1}=\frac{1}{cc}\left[\begin{array}{cc} M_{yy} & M_{xy}\\ M_{xy} & M_{xx} \end{array}\right]$$

$$cc=M_{xx}M_{yy}-M_{xy}{}^{2}$$
